# White matter connections of the inferior parietal lobule: A study of surgical anatomy

**DOI:** 10.1002/brb3.640

**Published:** 2017-03-08

**Authors:** Joshua D. Burks, Lillian B. Boettcher, Andrew K. Conner, Chad A. Glenn, Phillip A. Bonney, Cordell M. Baker, Robert G. Briggs, Nathan A. Pittman, Daniel L. O'Donoghue, Dee H. Wu, Michael E. Sughrue

**Affiliations:** ^1^ Department of NeurosurgeryUniversity of Oklahoma Health Sciences CenterOklahoma CityOKUSA; ^2^ Department of Cell BiologyUniversity of Oklahoma Health Sciences CenterOklahoma CityOKUSA; ^3^ Department of Radiological SciencesUniversity of Oklahoma Health Sciences CenterOklahoma CityOKUSA

**Keywords:** anatomy, angular, DTI, gyrus, inferior parietal, supramarginal, tractography, white matter

## Abstract

**Introduction:**

Interest in the function of the inferior parietal lobule (IPL) has resulted in increased understanding of its involvement in visuospatial and cognitive functioning, and its role in semantic networks. A basic understanding of the nuanced white‐matter anatomy in this region may be useful in improving outcomes when operating in this region of the brain. We sought to derive the surgical relationship between the IPL and underlying major white‐matter bundles by characterizing macroscopic connectivity.

**Methods:**

Data of 10 healthy adult controls from the Human Connectome Project were used for tractography analysis. All IPL connections were mapped in both hemispheres, and distances were recorded between cortical landmarks and major tracts. Ten postmortem dissections were then performed using a modified Klingler technique to serve as ground truth.

**Results:**

We identified three major types of connections of the IPL. (1) Short association fibers connect the supramarginal and angular gyri, and connect both of these gyri to the superior parietal lobule. (2) Fiber bundles from the IPL connect to the frontal lobe by joining the superior longitudinal fasciculus near the termination of the Sylvian fissure. (3) Fiber bundles from the IPL connect to the temporal lobe by joining the middle longitudinal fasciculus just inferior to the margin of the superior temporal sulcus.

**Conclusions:**

We present a summary of the relevant anatomy of the IPL as part of a larger effort to understand the anatomic connections of related networks. This study highlights the principle white‐matter pathways and highlights key underlying connections.

## 1 Introduction

Advances in neuroscience have created an emphasis on network connectivity in understanding brain function (Wang et al., 2015). Interest in the function of the inferior parietal lobule (IPL) has resulted in increased understanding of its involvement in visuospatial and cognitive functioning, and its role in semantic networks. The language network is thought to be divided across two pathways, often referred to as the dual‐streams model, wherein one pathway is responsible for conceptual‐semantic systems, and another distinct pathway is responsible for motor‐articulatory systems (Hickok & Poeppel, 2015). A ventral pathway projects within the superior temporal gyrus to link sound to meaning (Binder & Desai, 2011; Crinion, Warburton, Lambon‐Ralph, Howard, & Wise, 2006; Scott, Blank, Rosen, & Wise, 2000), and a dorsal pathway projects anteriorly within the frontal lobe to motor and premotor areas to integrate articulatory information (Binder, 2015; Pulvermuller et al., 2006; Saur et al., 2008; Warren, Wise, & Warren, 2005). Given its anatomic location, the IPL is uniquely situated at the crossroads of many important neurological processes, which may explain the variety of deficits patients may experience following stroke or surgery in this area.

Over the last several years, the major cortico‐cortical pathways associated within these regions have become increasingly well‐characterized (Chang, Raygor, & Berger, 2015). This includes the superior longitudinal fasciculus (SLF), inferior fronto‐occipital fasciculus (IFOF), arcuate fasciculus (Johnson‐Frey, Newman‐Norlund, & Grafton, 2005), and the inferior longitudinal fasciculus (ILF), among others (Tremblay, St. Dick, & Small, 2011). The middle longitudinal fasciculus (MdLF) is less characterized anatomically and functionally, but is involved in the semantic network (Caverzasi et al., 2015). Each of these bundles likely participates in normal cognitive processes, though the exact process by which information flows between relevant cortices is not well understood.

In this study, we sought to derive clinically actionable knowledge of the relationship between gyri of the IPL and their underlying major white‐matter bundles. We used diffusion tractography to characterize the macroscopic connectivity of the networks associated with the IPL, followed by gross anatomical dissection as ground truth. This is the first study that characterizes the nuanced anatomy of white matter connections between associated cortical areas in a way that may be of relevance to neurosurgeons attempting to preserve the neuronal networks dependent on connections in this region of the brain.

## 2 Methods

### 2.1 Tractography

Publicly available imaging data from the Human Connectome Project (Van Essen et al., 2013) were obtained for this study (http://humanconnectome.org, release Q3). Imaging was analyzed from 10 unrelated, healthy adult subjects (HCP Subject IDs: 100307, 103414, 105115, 110411, 111312, 113619, 115320, 117122, 118730, 118932). A multishell diffusion scheme was used, with *b*‐values of 990, 1,985, and 2,980 s/mm^2^. Each *b*‐value was sampled in 90 directions. The in‐plane resolution was 1.25 mm. The slice thickness was 1.25 mm. The diffusion data were reconstructed using generalized q‐sampling (GQI) imaging (Yeh, Wedeen, & Tseng, 2010) with a diffusion sampling length ratio of 1.25.

Following registration to MNI space, tractography with GQI (Yeh et al., 2010) was performed in DSI studio (http://dsi-studio.labsolver.org) using a region of interest approach (ROI) to initiate the fiber tracking from a user‐defined seed region (Martino et al., 2013). A two‐ROI‐approach (Kamali, Sair, Radmanesh, & Hasan, 2014) was used to isolate tracts and all were tested for reproducibility. Dissecting in MNI space allowed for ready assessment of variability among subjects. Voxels within each ROI were automatically traced with a maximum angular threshold of 45°. When a voxel was approached with no tract direction or a direction greater than 45°, the tract was halted. Tractography was stopped after reaching a length of 450 mm. In some instances, exclusion ROIs were placed to exclude spurious tracts or tracts not involved in the network of interest. Dissections proceeded systematically anteriorly to posteriorly along the continuation of the Sylvian fissure for the supramarginal gyrus (SMG; Ribas, 2010), and along the continuation of the superior temporal sulcus (STS) for the angular gyrus (AG; Naidich, Valavanis, & Kubik, 1995; Seghier, 2013).

### 2.2 Postmortem dissection

To validate tractography results we sought to demonstrate the location of major tracts connecting to the IPL with gross anatomical dissections as ground truth (Burks et al., 2016; Catani et al., 2012). Postmortem dissections were performed using a modified Klingler technique (Koutsarnakis, Liakos, Kalyvas, Sakas, & Stranjalis, 2015). Ten hemispheres were used for this study, obtained from our institution's Willed Body Program with approval of the state's anatomical board. All specimens were donated by individuals who died from causes unrelated to intracranial pathology. The cadaveric brains were obtained after standard embalming and further fixed in 10% formalin for at least 3 months after removal from the cranium. Up until the time of dissection, the pia‐arachnoid membrane was left attached.

After fixation with formalin, specimens were rinsed with water for 2 days, and then frozen at −10°C for 8 hr to disrupt the white matter. After thawing, dissection of the “freeze‐fractured” specimens began with removal of meninges and identification of cortical anatomy, including gyri and sulci. Relevant cortical areas were identified first. Starting superficially they were then pealed back to reveal white‐matter areas of interest, and care was taken to leave cortical areas corresponding to white‐tracts of interest intact to preserve their relationship with one another. Tracts were dissected with blunt instruments to avoid disrupting the natural tract anatomy with care taken not to create spurious tracts. Photographs were taken at each stage in the dissection, and spatial relationships were recorded. All SMG and AG tracts were dissected in both hemispheres.

### 2.3 Definition of anatomic boundaries of the angular and supramarginal gyri

Uniform anatomic boundaries were applied to our dissections and used for manually generated ROIs in fiber tracking. At the termination of the Sylvian fissure, the sulcus divides into superior, posterior, and horizontal rami. The posterior ramus continues posteriorly and superiorly a short distance before terminating, forming the distinctive upside down “U” of the SMG. Similarly, the STS, formed at the junction of the superior and middle temporal gyri, is traced to its termination in the AG. The morphology of the posterior STS is variable across individuals (Segal & Petrides, 2012), and in some instances the AG is identified by its relationship with the SMG. The SMG is bounded anteriorly by the postcentral gyrus, superiorly by the intraparietal sulcus (formed by junction with the superior parietal gyrus), and posteriorly by the intermediate sulcus of Jensen (formed at the junction with the AG), which can be a branch of the intraparietal sulcus or, in some cases, of the STS. The AG is bounded superiorly by the intraparietal sulcus, and posteriorly and inferiorly by the occipital lobe (Hodgetts et al., 2015).

### 2.4 Statistical analysis

Continuous variables were reported as means with standard deviations. Categorical variables were reported using frequencies and percentages. A Mann–Whitney *U*‐test was used to determine the relationship between an independent variable (hemisphere side) and continuous variables (distances measured between the termination of the Sylvian fissure and the SLF, and distances measured between the STS and the arcuate fasciculus). Other calculated means were compared using an unpaired *t* test.

A *p* value ≤.05 was considered statistically significant. All data analyses were completed using SPSS (Version 22; IBM Inc. New York, NY, USA).

## 3 Results

### 3.1 Local connections

**Figure 1:**
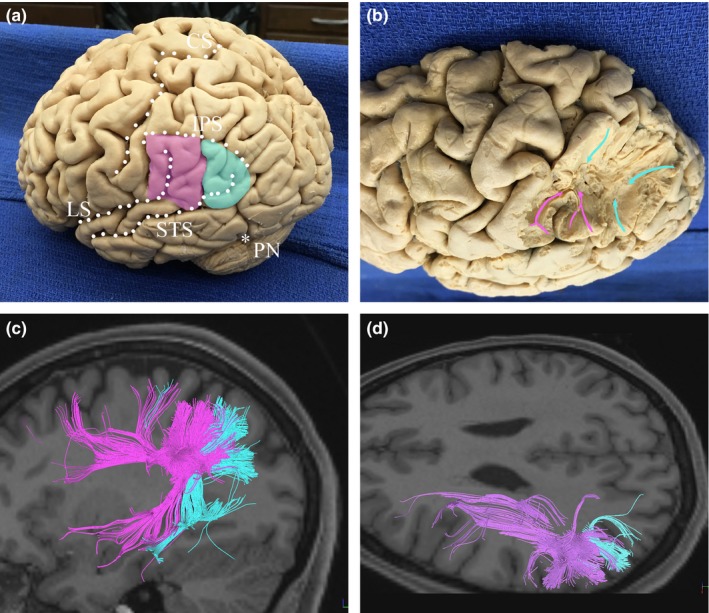
Superficial anatomy of the inferior parietal lobule (IPL). (a) The supramarginal gyrus (SMG) loops around the postero‐superior extension of the lateral sulcus (i.e., the Sylvian fissure). The angular gyrus (AG) similarly wraps around at the parietal extension of the STS. (b) Fiber dissection revealing the superficial connections of the SMG and AG. These tracts primarily facilitate local and intragyral connectivity, taking an oblique angle from the cortical surface. Some fibers from the SMG and AG travel superiorly to the superior parietal lobule (SPL). (c, d) Tractography demonstrating the orientation of superficial fibers from the IPL. Lateral (c) and supero‐lateral (d) views illustrate the fronto‐occipital and temporo‐occipital orientations of fibers connecting to the SMG, and the temporo‐occipital orientation of fibers connecting to the AG. Pink: SMG, fibers connecting to the SMG; Blue: AG, fibers connecting to the AG; IPS, intraparietal sulcus; LS, lateral sulcus (or Sylvian fissure); PN, preoccipital notch; STS, superior temporal sulcus

**Figure 2:**
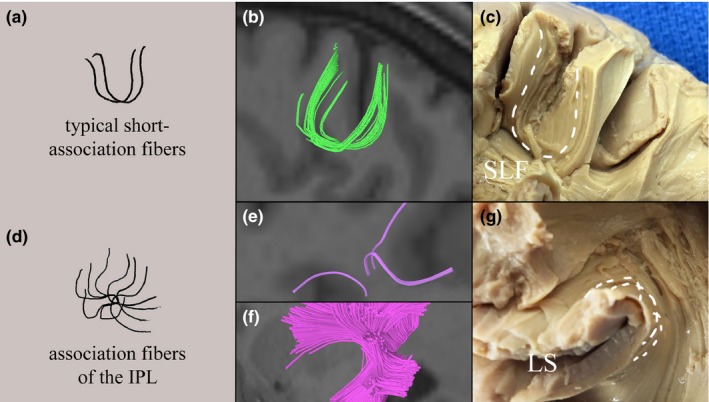
Short association fibers of the parietal lobe. Short association fibers of the cerebrum have a variable morphology, but tend to be “U‐shaped” as they connect adjacent gyri, as illustrated by (a). Short association fibers of the superior parietal lobule are shown with tractography (b) and gross dissection (c). Short association fibers of the supramarginal gyrus (SMG) have a distinct morphology (d), as its short association fibers must negotiate the lateral sulcus. As fiber bundles leave the major white‐matter pathways to connect to the cortical surface, they become intertwined and twist slightly as they course superficially. The hydra‐like shape of these fibers is illustrated by tractography showing isolated fibers in (e) and fibers of the SMG in total (f). Gross dissection of association fibers beneath the cortex of the SMG is shown in (g). LS, lateral sulcus; SLF, superior longitudinal fasciculus

Immediately below the cortical surface of the supramarginal and angular gyri (SMG and AG, respectively) are the short connections of the IPL. The preoccipital notch approximates the boundary of the parietal and occipital lobes, and removal of the superficial white matter along this line posteriorly and superiorly along the intraparietal sulcus allows for visualization of the local connections of the AG and SMG (Figure 1). Some short association fibers (also called “U” fibers; Song, Chang, Petty, Guidon, & Chen, 2014) course superiorly, connecting to the superior parietal gyrus. Short association fibers at the termination of the lateral sulcus have a distinct, hydra‐like morphology, shown in Figure 2. Tractography demonstrates that local bundles of the AG tend to remain posterior to bundles of the SMG.

### 3.2 Long‐range connections between the IPL and frontal lobe

**Figure 3:**
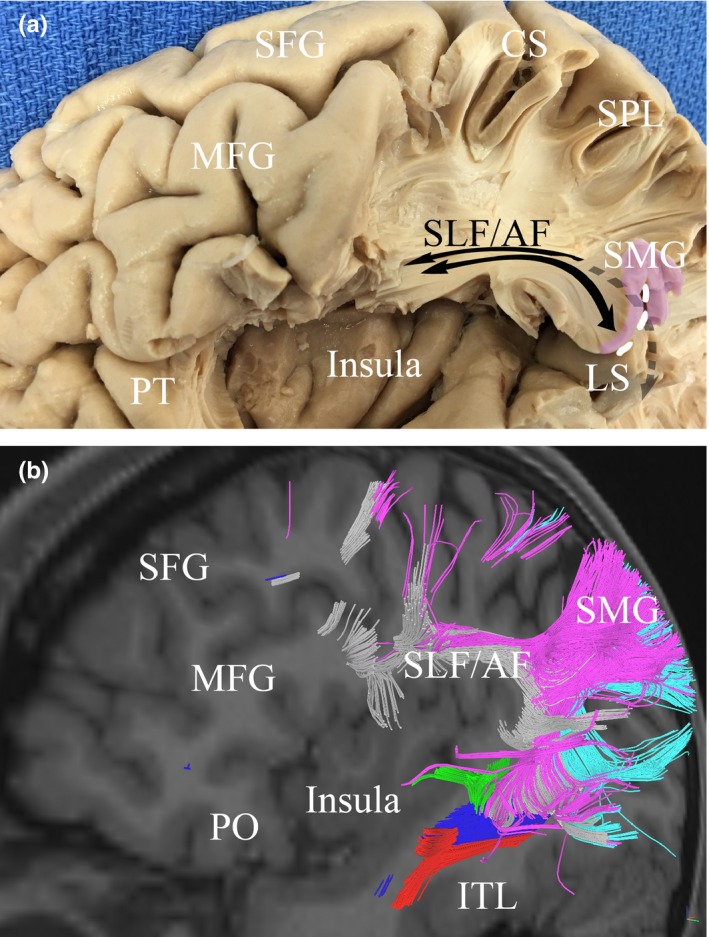
The relationship between the inferior parietal lobule (IPL) and the insula. Gross dissection (a) and tractography (b) illustrate the proximity of the insula to fibers connecting the IPL to the frontal lobe. Fibers connecting to frontal regions and the AG curve with the arcuate fasciculus around the posterior edge of the insula before becoming part of the superior longitudinal fasciculus (SLF), passing directly superior to the insula. Similarly, fibers connecting to frontal regions and the SMG join the SLF to course antero‐posteriorly just above the insula. Red, inferior longitudinal fasciculus; Blue, inferior fronto‐occipital fasciculus; Green, middle longitudinal fasciculus; Purple, SMG/fibers connecting to the SMG; Light Blue, AG /fibers connecting to the AG; white, superior longitudinal fasciculus; CS, central sulcus; ITG, inferior temporal gyrus; LS, lateral sulcus; MFG, middle frontal gyrus; PO, pars orbitalis; SFG, superior frontal gyrus; SLF/AG, superior longitudinal fasciculus/arcuate fasciculus; SMG, supramarginal gyrus

The SLF conveys fibers to terminations throughout the inferior and middle frontal gyrus visible in gross dissection, though tractography reveals most bundles originating in the IPL terminate in the posterior portion of the inferior frontal gyrus. Fibers coming from the SMG and AG join the SLF near the superior aspect of the termination of the Sylvian fissure. Beneath the SMG, fibers of the AG join the AF. Fibers from the AG course to the SLF posterior to fibers from the SMG, and within the SLF run inferior to fibers from the SMG. The anterior AF begins to curve inferiorly after passing above the insula, as shown in Figure 3.

### 3.3 Temporal connections with the IPL

**Figure 4:**
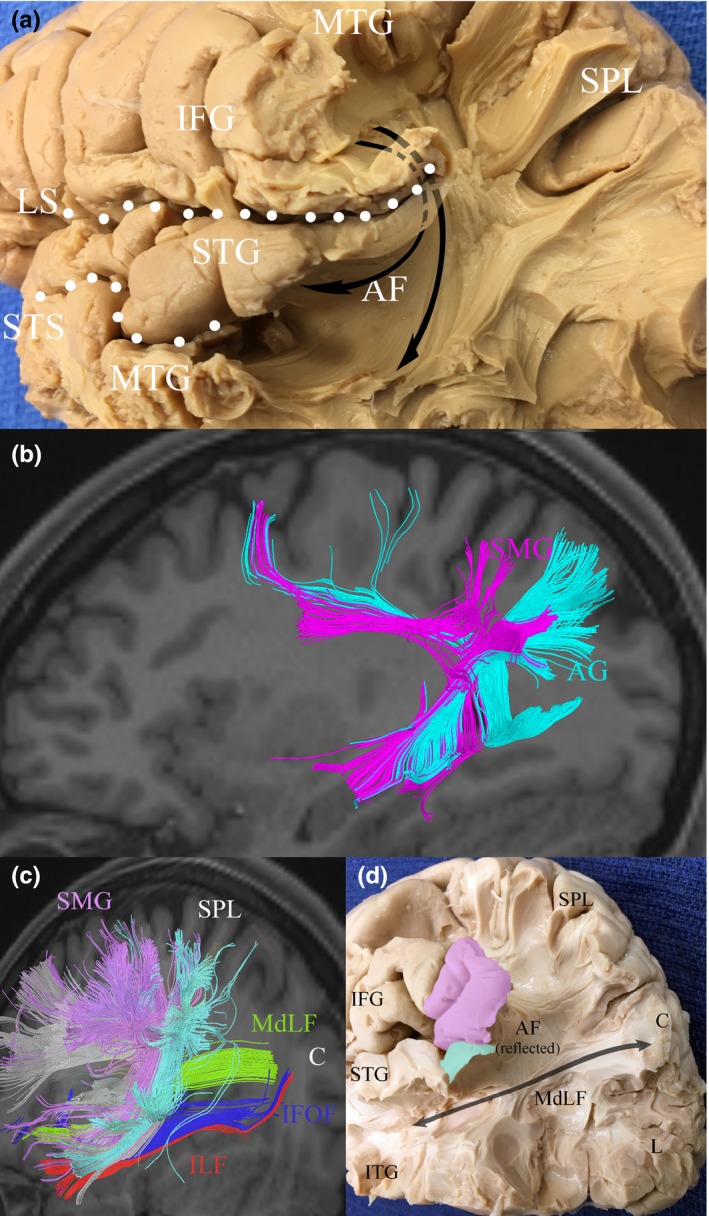
White matter anatomy of the inferior parietal lobule (IPL). The AF courses beneath the SMG around the termination of the Sylvian fissure to connect frontal areas to the posterior MTG and STG as shown in (a) and (b). The SMG connects with frontal areas by coursing with SLF III, and the AG connects with frontal areas via SLF II, also shown in (b). Some fibers from the SMG and AG travel a short distance to the temporal lobes with the AF. Once in the temporal lobe, these fibers travel parallel to the MdLF (b and c). The MdLF is shown connecting the STG to the cuneus in (d), passing deep to the IPL and AF. AF, arcuate fasciculus; AG, angular gyrus; C, cuneus; IFG, inferior frontal gyrus; IFOF, inferior fronto‐occipital fasciculus; ILF, inferior longitudinal fasciculus; L, lingual gyrus; LS, lateral sulcus; MdLF, middle longitudinal fasciculus; MTG, middle temporal gyrus; SLF, superior longitudinal fasciculus; SMG, supramarginal gyrus; STG, superior temporal gyrus

**Figure 5:**
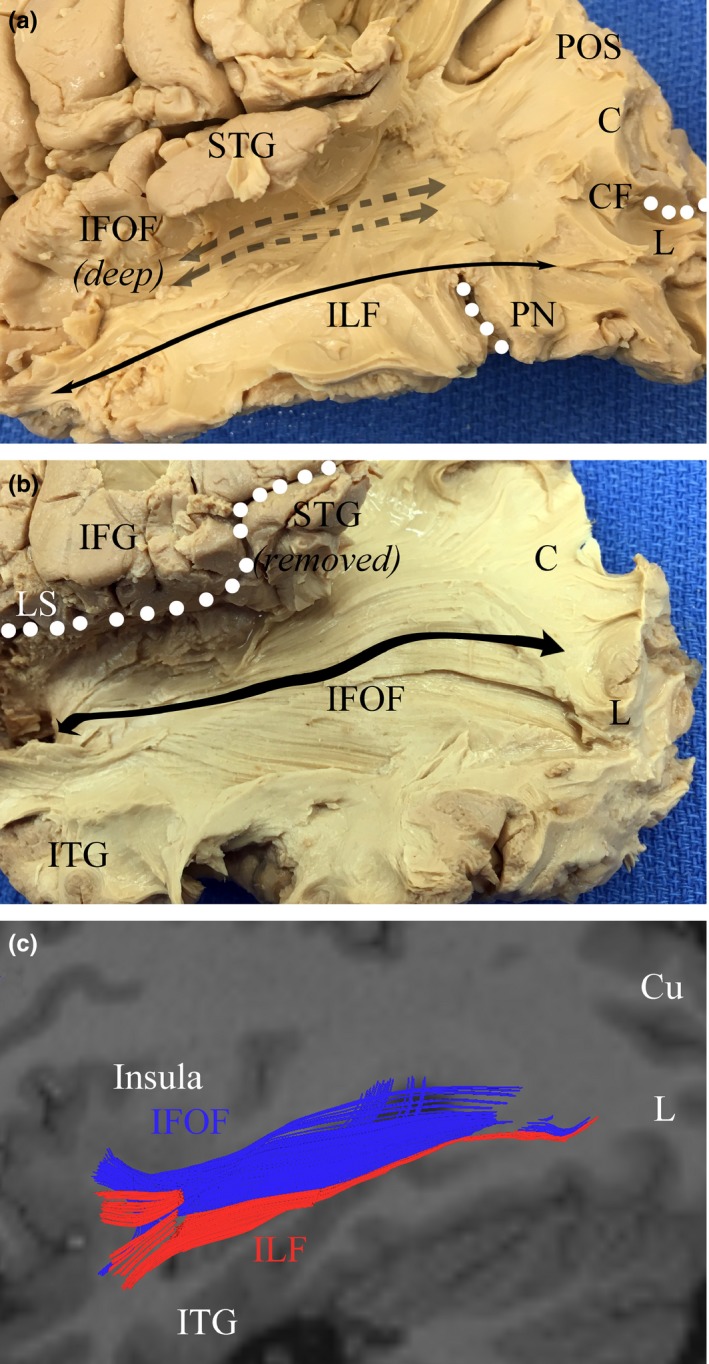
White‐matter pathways deep to the inferior parietal and posterior temporal lobes. Running medial and inferior to the fibers of the middle longitudinal fasciculus (MdLF) are the bundles of the inferior longitudinal fasciculus (ILF), which travel with fibers connecting the inferior parietal lobule to the ITG. The ILF is shown by fiber dissection in (a). The IFOF runs from prefrontal regions in the frontal lobe into the medial temporal lobe inferior to the insula, before coursing medial and parallel to the MdLF and terminating throughout the occipital lobe, as shown in (b). These tracts are shown by tractography in (c). Blue, IFOF; Red, ILF; Cu, cuneus; CF, calcarine fissure; IFG, inferior frontal gyrus; IFOF, inferior fronto‐occipital fasciculus; ITG, inferior temporal gyrus; L, lingual gyrus; LS, lateral sulcus; PN, preoccipital notch; POS, parieto‐occipital sulcus; STG, superior temporal gyrus; MdLF, middle longitudinal fasciculus

The MdLF courses posteriorly through the temporal lobe deep to the STS. The tract begins anteriorly within Heschl's gyrus, coursing with major fiber projections of the SMG, as shown in Figure 4. Posteriorly, radiations to the AG are present along the inferior margin of the STS. The MdLF passes just deep to the arcuate fasciculus, which forms a large, overlying “C”, carrying fibers of the SLF inferiorly. This intersection occurs beneath the termination of the STS. Some fibers of the IPL continue inferiorly to course with the ILF to terminate in the antero‐lateral portions of the middle and inferior temporal gyri. Fibers of the uncinate fasciculus terminate in the antero‐medial portions of the superior and middle temporal gyri. Fibers of the ILF and MdLF remain in parallel, lateral to fibers of the IFOF throughout (see Figure 5).

**Figure 6:**
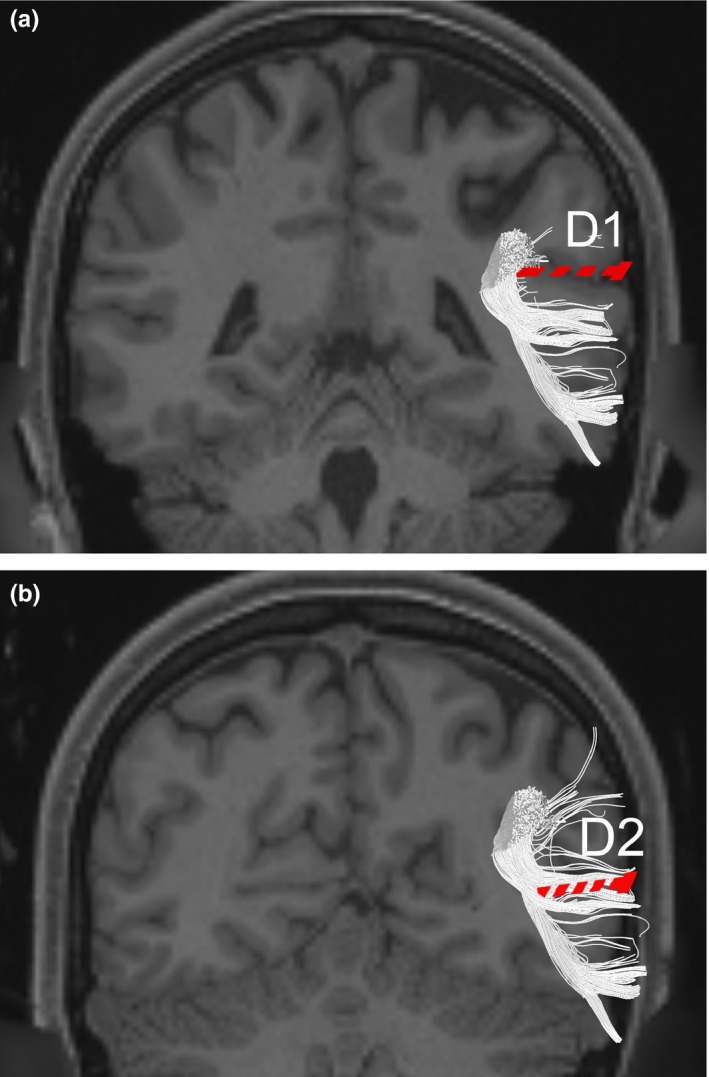
Defining D1 and D2. T1‐weighted coronal MR imaging illustrating D1, which was the shortest distance between the termination of the Sylvian fissure and the superior longitudinal fasciculus, is shown in (a). A slice taken slightly posterior to (a), illustrating D2, which was the shortest distance between the termination of the superior temporal sulcus and the arcuate fasciculus, is shown in (b). Both distances were measured in imaging and anatomical specimens. White: superior longitudinal fasciculus/arcuate fasciculus; Red: distance measured

Shortest distances were measured between the cortical surface at the termination of the Sylvian fissure and the SLF (D1), and between the cortical surface at the termination of the STS and the arcuate fasciculus (D2; illustrated in Figure 6). These distances give the approximate distance fibers of the SMG and AG travel from major associated white tracts before terminating near the cortical surface, and are presented in Table 1. These measurements are thus also a depth approximation of the association fibers of the IPL (which remain superficial to major tracts), and represent distance between cortical landmark and major white tract. D1 mean by tractography was 19 ± 2 mm on the right and 21 ± 2 mm on the left (*p *=* *.22); D2 mean by tractography was 18 ± 2 mm on the right and 19 ± 4 mm on the left (*p *=* *.83). D1 mean by fiber dissections was 19 ± 2 mm on the right and 21 ± 3 mm on the left (*p *=* *.42); D2 mean by fiber dissections was 16 ± 2 mm on the right and 16 ± 2 mm on the left (*p *=* *.52). There was no significant difference between distances calculated in tractography and fiber dissections for D1 (*p *=* *.45), but there was a significant difference in distances calculated for D2 (*p *=* *.03). This is shown in Table 2.

**Table 1:** Spatial relationship of the inferior parietal lobule to major white‐matter pathways

Subject		Tractography D1 (mm)	Tractography D2 (mm)	Fiber dissections D1 (mm)	Fiber dissections D2 (mm)
1	R	22	17	23	14
	L	19	14	22	16
2	R	17	17	16	18
	L	19	19	21	18
3	R	18	18	22	17
	L	23	24	24	14
4	R	20	21	17	17
	L	20	21	18	14
5	R	18	18	20	15
	L	21	16	21	16
Mean	R	19 ± 2	18 ± 2	19 ± 2	16 ± 2
	L	21 ± 2	19 ± 4	21 ± 3	16 ± 2
*p*	—	.22	.83	.42	.52

D1, nearest distance between the termination of the Sylvian fissure and the superior longitudinal fasciculus; D2, nearest distance between termination of the superior temporal sulcus and the arcuate fasciculus. R, right hemisphere; L, left hemisphere.

**Table 2:** Differences between measurements recorded in tractography and fiber dissections

Parameter	Mean	*p*
D1		
Tractography	20 ± 2	.45
Fiber dissections	20 ± 3	
D2		
Tractography	16 ± 2	.03
Fiber dissections	18 ± 3	

D1, nearest distance between the termination of the Sylvian fissure and the superior longitudinal fasciculus; D2, nearest distance between termination of the superior temporal sulcus and the arcuate fasciculus.

## 4 Discussion

Connectomic approaches to the brain have focused on mapping the cerebrum as a single network in full microstructural detail (Jbabdi, Sotiropoulos, Haber, Van Essen, & Behrens, 2015; Van Essen et al., 2013). In contrast, we have focused on macroconnectomic descriptions of medium and large fiber‐tracts and their functional and anatomic relationship with the SMG and AG to characterize surgically relevant anatomy. Using tractography and gross fiber‐tract dissection as ground truth, we have described the white matter connections of the IPL with respect to major underlying white‐matter pathways, and have further characterized the MdLF.

As a more detailed understanding of the functional mechanisms of the IPL develops, the ability to identify these tracts intraoperatively is likely to be of significance. Presenting information that is readily applied to current operative techniques is our goal. Additionally, manual seeding of the regions identified using D1 and D2 could further be used to identify the major cortical connections of the semantic network in diffusion tensor imaging (DTI) integrated, image‐guided procedures.

Our study differs from earlier studies (Caspers et al., 2006, 2011), which sought to understand IPL connectivity through tractography and microarchitectural analysis. This study included a parcellation‐based connectivity analysis, the results of which were similar to earlier studies. However, the primary focus of this work was not to quantify connectivity, but rather to characterize the anatomy of the white‐matter bundles responsible for observed connectivity. This anatomy has not been previously described. Although we chose to focus on anatomical and functional relationships, at this time, it is difficult to hypothesize which functions are paired with particular tracts. However, we offer the most likely models based on our evidence that these tracts make up the majority of connectivity of the IPL.

Earlier works have studied IPL connectivity in humans in relation to macaques, because most of what was initially known about IPL connectivity came from macaque injection studies (Mars et al., 2011). Though studies have suggested the IPL has regions and functions that are distinct in humans, anatomic connectivity informs functional segregation of cortical areas (Averbeck, Battaglia‐Mayer, Guglielmo, & Caminiti, 2009; Passingham, Stephan, & Kotter, 2002). Through connectivity analysis using tractography, these authors have shown that functional connectivity is mostly conserved in humans.

The results of our connectivity analysis performed solely in humans, for characterizing key white‐matter fiber bundles, were mostly in agreement with principle earlier studies. Caspers et al. (2011) and Mars et al. (2011) previously noted connections to premotor and prefrontal areas, and Ruschel et al. (2014) observed key tracts connecting with the superior parietal lobule (SPL) and ITG. These patterns are consistent with our findings. Ruschel et al. (2014) also described connectivity with the ITG, which was not seen in our tractography.

### 4.1 Key structures

#### 4.1.1 Gross organizational principles

The AG, located in the posterior IPL, meets the SMG at the descending portion of the intermediate sulcus of Jensen. The posterior boundary of the AG is adjacent to the dorsal anterior occipital sulcus (Seghier, 2013). The anterior‐most portion of the intraparietal sulcus has been associated with ventral premotor areas, the middle frontal gyrus, and insula, whereas the posterior‐most portion is linked with extrastriate visual areas (Uddin et al., 2010). The anterior AG is linked to the ventral premotor area and ventrolateral prefrontal cortex, whereas the posterior AG is associated with ventromedial prefrontal cortex, posterior cingulate, and hippocampus (Uddin et al., 2010). We observed direct connections between the SMG and AG by way of short association fibers (U‐fibers). We also observed U‐fibers connecting both the SMG and AG to the SPL. Our study also further confirms direct connections to the posterior portion of the inferior frontal gyrus (IFG) and extensively throughout the posterior temporal lobe. A summary of connectivity is given in Table 3.

**Table 3:** Gross connectivity^a^

Gyrus	Target	Connecting pathway
SMG	MTG	AF/MdLF
	STG	AF/MdLF
	MFG	SLF
	AG	Local
	SPL	Local
	PSC	Local
	PMC	SLF
	PO	SLF
AG	SPL	Local
	MTG	AF
	LO	ILF
	PSC	SLF

AF, arcuate fasciculus; AG, angular gyrus; CU, cuneus; ILF, inferior longitudinal fasciculus; LO, lateral occipital; MFG, middle frontal gyrus; MTG, middle temporal gyrus; PMC, primary motor cortex; SLF, superior longitudinal fasciculus; SMG, supramarginal gyrus; SPL, superior parietal lobule; STG; superior temporal gyrus; PO, pars orbitalis; PSC, primary sensory cortex.

a Based on tractography and fiber‐tract dissection.

The novelty in this study is in the surgical/clinical application of this information. In presenting a surgical atlas, we outline several tracts and salient features of these tracts not found elsewhere. In provided D1 and D2 in Table 2, we offer a precise anatomical relationship of these fiber bundles and offer a method of approximating their location in the operating room. From this information, we can conclude that the bundles connecting to major white‐matter tracts such as the SLF/ALF, do so at approximately a thumb's breadth from the cortical landmark (the termination of the Sylvian fissure for D1; the termination of the superficial temporal sulcus for D2). Such qualitative data with direct application in the operating room has not been previously published with respect to the IPL.

#### 4.1.2 Long‐association fiber bundles: the inferior longitudinal fasciculus and middle longitudinal fasciculus

In our analysis, both the MdLF and ILF convey fiber bundles of the IPL to the temporal lobe. Our dissection revealed projections of the AG and SMG along the inferior margin of the STS that eventually joined the MdLF or ILF. As previously described by others (Catani, Jones, Donato, & Ffytche, 2003), the ILF is a direct occipito‐temporal pathway distinct from the optic radiations and the indirect pathway formed by the short association fibers along the same route. The ILF has been implicated in other studies as connecting the lateral occipital and temporal areas to the IPL, and these tracts have been shown to originate from the caudal IPL (Caspers), which was consistent with our results.

Earlier authors have described a MdLF, which extends from the AG, deep to the arcuate fasciculus (Johnson‐Frey et al., 2005), along the course of the STG to the temporal pole (Menjot de Champfleur et al., 2013). Makris et al. (2009) have proposed the bundle serves some combination of roles traditionally attributed to the AF, serving as a route between the AG and STG. Others have suggested participation of the tract in dorsal and ventral processing streams (Saur et al., 2008). Other studies have suggested the presence of this tract, but we propose a definitive location of this tract within Heshl's gyrus connecting to the precuneus passing lateral to the bundles of the arcuate connecting to the temporal lobe. However, outstanding questions remain regarding function (Dick & Tremblay, 2012). However, in light of shifting insights into the brain as a parallel processing network, it seems likely the tract has a role in cognition even if not as a principle pathway (De Witt Hamer, Moritz‐Gasser, Gatignol, & Duffau, 2011).

Our study additionally highlights the convergence of major white‐matter pathways beneath the IPL. The temporal‐parietal junction, at the anterior margin of the AG, has been characterized as an integrating area of multiple, intersecting pathways in the extended semantic network (Binder & Desai, 2011; Jouen et al., 2015). Martino and colleagues previously described the area deep to this region as the temporo‐parietal fiber intersection area, which includes the SLF, AF, IFOF, ILF, MdLF, optic radiations, and tapetum (Martino et al., 2013). In other words, the IPL has significant connectivity with all major semantic areas of the brain.

#### 4.1.3 Superior longitudinal fasciculus and arcuate fasciculus connectivity

Both gyri of the IPL convey fibers to frontal and prefrontal areas. We observed fibers connecting to the SMG and joining the SLF just medial and anterior to the terminus of the Sylvian fissure. These fibers from the SMG also incorporated descending fibers from primary sensory cortex superiorly and the AG posteriorly into the AF. Other studies have suggested a similar pattern of connectivity to the SPL (Kamali, Sair, et al., 2014) that utilizes the AF in connecting to the temporal gyri (Kamali, Flanders, Brody, Hunter, & Hasan, 2014). In agreement with previous studies, we noted that frontal connections from the parietal lobe extend via the AF to branch into the central premotor cortex to Brodmann area (BA) 6 (i.e., supplemental motor area) before turning anteriorly below the IFG (Ramayya, Glasser, & Rilling, 2010). The language pathway connecting the posterior STG and posterior MTG beneath the IPL to BA 44 and 45 of the IFG reported by others (Catani, Jones, & ffytche, 2005) was well‐visualized throughout our study.

Earlier studies have classified the AF differently in the literature. Catani et al. (2005) designate the aforementioned direct pathway, as well as an indirect pathway connecting temporal and parietal areas, and parietal and frontal areas. These segments link Wernicke territory to IPL and IPL to posterior Broca territory, respectively (Schmahmann & Pandya, 2006; Urbanski et al., 2011). It has been suggested that the so‐called anterior segment of AF actually corresponds to the third branch of the SLF (Duffau, Moritz‐Gasser, & Mandonnet, 2014; Schmahmann & Pandya, 2006; Yagmurlu, Middlebrooks, Tanriover, & Rhoton, 2015), which associates rostral IPL, from the SMG, to BA 44 (Frey, Campbell, Pike, & Petrides, 2008). In our imaging, the posterior SLF connects the posterior MTG with the AG at the caudal end of the IPL. Here, the AF underlies the AG where temporal cortex fibers also converge (Martino et al., 2013).

In contrast to the anterior/posterior description of the indirect AF, Yagmurlu and colleagues characterize the tract's relationship with IPL by designating ventral (corresponding to classic AF) and dorsal AF segments, both of which terminate in the frontal lobe (Yagmurlu et al., 2015). They found the ventral AF was found to run deep to the lower part of the SMG (BA 40), linking middle and posterior STG to the IFG and ventral premotor cortex (Catani et al. (2005)). The dorsal AF segment, in contrast, runs deep to the lower part of the AG (BA 39; Yagmurlu et al., 2015).

Our study offers support for the structure initially described by Catani and colleagues, that a direct pathway links the STG and IFG, with an indirect, overlying pathway containing descending fibers from the IPL (see Figure 3). The AF fits an anterior/posterior model based on observed tract morphology and connectivity. Furthermore, the SLF and AF do not appear to be distinct structures. Our experience tracking these structures indicates fibers isolated to the AF are present, though surrounded by bundles continuous with the SLF, and are virtually inseparable in anatomic specimens.

#### 4.1.4 Inferior frontal‐occipital fasciculus connectivity

The IFOF has been a suspected link to frontal areas involved in semantic processing (Motomura et al., 2014). A second layer of the IFOF, the deep ventral segment, connects to posterior and basal temporal regions as well as the posterior occipital gyrus (Sarubbo, De Benedictis, Maldonado, Basso, & Duffau, 2013). Other authors have suggested the IFOF receives contributions from the AG (Uddin et al., 2010), though we did not ascertain this in our tractography. Based on our study, it seems likely any processing from the IPL reaches prefrontal regions through indirect, rather than direct, pathways.

Of note, previous authors have suggested a lack of evidence for a superior fronto‐occipital fasciculus (Meola, Comert, Yeh, Stefaneanu, & Fernandez‐Miranda, 2015; Ture, Yasargil, & Pait, 1997). Consequently, some authors have used occipito‐frontal fasciculus in place of IFOF (Makris et al., 2007; Reuter et al., 2009). In this study, we have maintained the conventional terminology to avoid confusion.

### 4.2 Functional significance

What is known about the IPL comes from stroke studies, tumor surgery, and most recently, functional magnetic resonance imaging (fMRI). From fMRI, we understand that the IPL functions simultaneously with other cortices to perform certain neurological functions. The IPL is thought to be involved in semantic processing, including several uniquely human capabilities cite as below.

Semantic processing is the cognitive function of accessing knowledge learned from experience (Meteyard, Cuadrado, Bahrami, & Vigliocco, 2012). Areas involved in semantic processing include the IPL, prefrontal areas, inferior frontal gyrus, and middle temporal gyrus, among others, and are reliably conserved across individuals (Binder, Desai, Graves, & Conant, 2009). The IPL, ideally situated at the interface between several information convergence zones (Damasio, 1989), is thought to integrate conceptual tasks (Binder & Desai, 2011). This allows for efficient retrieval of knowledge and may explain many uniquely human capabilities (Binder et al., 2009).

#### 4.2.1 Semantic functioning: reading, writing, and phonological processing

The IPL has been implicated in writing and phonological processing networks. Along with the SPL, premotor areas, and supplemental motor areas, the IPL is activated during fMRI of writing tasks (Motomura et al., 2014). Fibers of the dorsal IFOF, important for reading and writing tasks, coordinate with the SLF to transmit word images between the frontal, parietal, and inferior temporal lobes. Damage to the left SLF in the region of the primary sensory area of the IPL has been shown to cause omission of graphemes (kana and kanji characters) within sentences, or dysgraphia, whereas apraxic dysgraphia usually occurs secondary to damage of the SPL (Shinoura et al., 2012). Interestingly, these areas are disproportionately large in humans compared to other primates (Orban, Van Essen, & Vanduffel, 2004).

White matter underlying the IPL is highly involved in the dorsal phonological system (Maldonado, Moritz‐Gasser, & Duffau, 2011), one of two distinct neural routes (the other being the ventral orthographic) involved in reading processes (Motomura et al., 2014). The left temporoparietal junction encompassing the AG and SMG, along with posterior STG and opercular part of Broca's area, are included in the dorsal route and function in word decoding (Motomura et al., 2014). Fiber tracts of the dorsal stream include the SLF and AF (Yagmurlu et al., 2015). Using direct electrostimulation, Maldonado and colleagues determined that horizontal SLF fibers connect SMG to ventral premotor cortex and underlie articulatory processes, whereas deep arcuate fibers are involved in phonological processing (Maldonado et al., 2011). They found the SLF and AF do not participate in language semantic processing (Maldonado et al., 2011).

#### 4.2.2 Spatial attention

The nondominant IPL and intraparietal fissure have been consistently implicated in spatial awareness (Roux et al., 2011). Awake mapping of supramarginal and angular areas have correlated stimulation with neglect (Talacchi et al., 2013). The SLF, in the region of the IPL, is thought to contribute to this function (Doricchi & Tomaiuolo, 2003). There is some evidence in the form of maximal rightward shifts on line bisection tasks with stimulation in this area (Thiebaut de Schotten et al., 2005). In the absence of damage to the IFOF and ILF, damage to the right SLF in the area of the IPL has also been shown to play a role in left spatial neglect (Shinoura et al., 2009).

While damage to the right IPL has long been known to cause hemi‐spatial neglect (Kravitz, Saleem, Baker, & Mishkin, 2011; Ramayya et al., 2010; Thiebaut de Schotten et al., 2005), the posterior SPL along with the SLF and IFOF have also demonstrated involvement in orienting spatial attention (Vallar et al., 2014). On the basis of the presence of extensive connectivity with the IPL, we suspect the SPL is involved in higher processing of functions attributed to the IPL.

#### 4.2.3 Tool use, gestured action, and working memory

The IPL conveys visuospatial, proprioceptive, and cognitive information to the premotor cortex via several distinct pathways in order to carry out tool‐use gestures (Ramayya et al., 2010). The posterior MTG and anterior SMG integrate ventral‐stream object recognition with dorsal‐stream localization, the anterior SMG and frontal lobe integrate gesture planning with the premotor cortex, and the connection between SMG and AG with the frontal lobe acts in spatial awareness (Ramayya et al., 2010). The dorsal stream, which includes the posterior half of the IPL (Sala‐Llonch, Palacios, Junque, Bargallo, & Vendrell, 2015), may be needed to process tools and manipulable objects as a specific category (Almeida, Mahon, Nakayama, & Caramazza, 2008). Comparison between macaques and human IPLs using fMRI and tractography found vast similarities in IPL organization, but the area responsible for tool observation was one of two areas unique to human IPL (Mars et al., 2011). More recent studies propose that the dorsal stream processes shape—specifically the elongated profile of many tools—as opposed to the object as a category in and of itself (Ludwig, Kathmann, Sterzer, & Hesselmann, 2015; Sakuraba, Sakai, Yamanaka, Yokosawa, & Hirayama, 2012).

The right IPL is activated during the maintenance period of visual working memory, in which the brain segregates information into domain‐specific regions according to cues such as color, location, and orientation (Passaro et al., 2013). Semantic tasks of object activation and location activation have been associated with the link between the lingual gyrus and IPL bilaterally (Passaro et al., 2013). The right IPL has also been shown to function in auditory spatial working memory tasks by monitoring and updating sound location (Alain, He, & Grady, 2008). The right IPL is thus critical in both auditory and visual location working memory, with an underlying white‐matter framework to support independent pathways (Sinnett, Juncadella, Rafal, Azanon, & Soto‐Faraco, 2007).

#### 4.2.4 Limitations

Any anatomical study represents a challenge as there are limitations with any specific technique. The smallest connections are not visible with gross dissection, and microconnectivity as demonstrated with tractography cannot be validated ex vivo. Thus, tractography is likely to be the best method of comparison to other studies. However, clinically actionable anatomy is the focus of this study, and as such, the tracts presented were identified with both techniques.

## 5 Conclusions

This study highlights the principle white‐matter pathways of the IPL and highlights key underlying connections. We present a summary of the relevant clinical anatomy for this region of the cerebrum as part of a larger effort to understand the network in its entirety.

## Conflict of Interest

The authors have no conflicts of interest or sources of funding to disclose.
